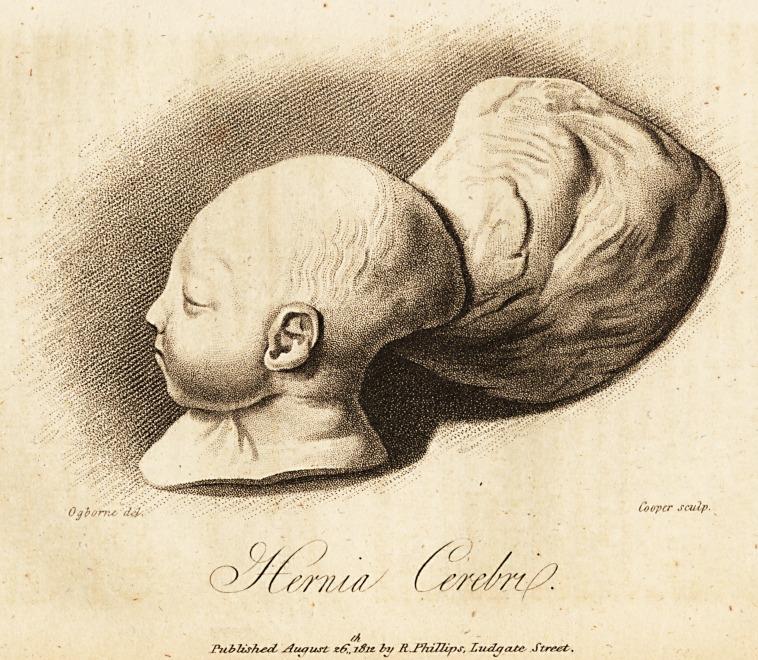# Account of a Hernia of the Brain

**Published:** 1812-09

**Authors:** 

**Affiliations:** Surgeon, Aberdeen


*For the Medical and Physical Journal.*

### *Account of a Hernia of the Brain. By* Mr. Hector, Surgeon, Aberdeen.*

(With a Plate.)

ON the 4th of November, 1811, I was called to a woman who had been in labor the two preceding days, with a midwife attending her.

Upon examination *per vaginam*, I ascertained that the pelvis of the woman was well formed, the *os uteri* dilated, but no part of the child could be felt: the next pain, however, brought within the reach of the fingers, a large soft tumor, the nature of which I could not then determine. As the pain increased, this tumor came down before the head,

---

* We are indebted to the records of the Blenheim-street Medical Society for the above history, and the cast from which the drawing was made.—Editors.

and

Ogborne del.　　　　Cooper sculp.

*Hernia Cerebri.*

Published August 26th, 1812 by R. Phillips, Ludgate Street.

and was evidently attached to it. Without much difficulty the child was born by the natural action of the uterus. It was a well-formed boy in every part, except the head, which was considerably smaller than common, with a large fleshy tumor attached to the occiput. The child lived twenty-four hours, which it passed in apparently great pain, crying almost incessantly.

In the presence of doctors More and Ogilvie, an examination of this tumor and of the head was made. An incision from the occiput to the extremity of the tumor discharged a quantity of extravasated blood, and exposed a portion of cerebrum, conjectured to be two-thirds of the whole brain; and of which the tumor was formed. Some of the convolutions were whole, but enlarged, and others appeared considerably contused. The whole was removed, with the sac formed of the common integuments, to examine the aperture in the cranium through which the cerebrum had passed. This was found to be a circle, the diameter of which was an inch and a third, with the edges, being soft and cartilaginous, turned outward.

From a fact related by the woman, there seems some probability that this disease was occasioned by a blow which the mother had received in the fifth month of her pregnancy.

## Figures and Tables

**Figure f1:**